# 2-((*E*)-{2-[(*E*)-2,3-Dihydroxy­benzyl­ideneamino]-5-methyl­phen­yl}iminiometh­yl)-6-hydroxy­phenolate

**DOI:** 10.1107/S1600536809029833

**Published:** 2009-08-08

**Authors:** Naser Eltaher Eltayeb, Siang Guan Teoh, Chin Sing Yeap, Hoong-Kun Fun, Rohana Adnan

**Affiliations:** a School of Chemical Science, Universiti Sains Malaysia, 11800 USM, Penang, Malaysia; b X-ray Crystallography Unit, School of Physics, Universiti Sains Malaysia, 11800 USM, Penang, Malaysia

## Abstract

The asymmetric unit of the title Schiff base compound, C_21_H_18_N_2_O_4_, consists of four independent zwitterions (*A*, *B*, *C* and *D*) with similar conformations. In each independent mol­ecule, the methyl group is disordered over two positions; the occupancies of the two positions are 0.819 (5) and 0.181 (5) in mol­ecule *A*, 0.912 (5) and 0.088 (5) in *B*, 0.734 (5) and 0.266 (5) in *C*, and 0.940 (6) and 0.060 (6) in *D*. The dihydroxy­phenyl and the hydroxy­phenolate rings in mol­ecule *A* form dihedral angles of 17.36 (12) and 13.30 (12)°, respectively, with the central benzene ring, whereas the respective angles for mol­ecules *B*, *C* and *D* are 30.22 (11)/7.46 (11), 35.26 (12)/11.01 (12) and 39.89 (12)/4.29 (12)°. In all independent mol­ecules, intra­molecular N—H⋯O and O—H⋯N hydrogen bonds generate *S*(6) ring motifs. The four independent mol­ecules are linked into two pairs, *viz. A*–*B* and *C*–*D*, by inter­molecular O—H⋯O hydrogen bonds. These pairs are linked into a two-dimensional network parallel to the *ab* plane by C—H⋯O hydrogen bonds. In addition, C—H⋯π and π–π [centroid–centroid distance = 3.5153 (14)–3.7810 (15) Å] inter­actions stabilize the crystal structure.

## Related literature

For the biological applications of Schiff base derivatives, see: Dao *et al.* (2000 ▶); Eltayeb & Ahmed (2005*a* ▶,*b* ▶); Karthikeyan *et al.* (2006 ▶); Sriram *et al.* (2006 ▶). For related structures, see: Eltayeb *et al.* (2007*a* ▶,*b* ▶, 2008 ▶). For hydrogen-bond motifs, see: Bernstein *et al.* (1995 ▶). For the stability of the temperature controller used for the data collection, see: Cosier & Glazer (1986 ▶). 
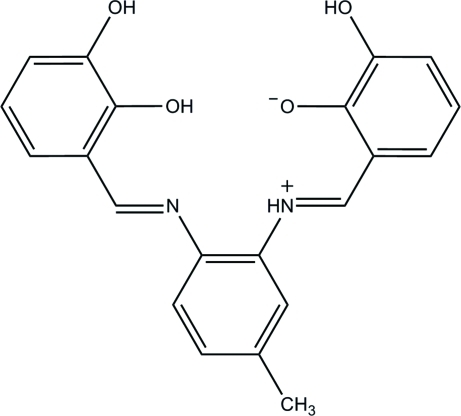


## Experimental

### 

#### Crystal data

C_21_H_18_N_2_O_4_
*M* *_r_* = 362.37
Monoclinic, 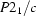

*a* = 17.0634 (2) Å
*b* = 21.3455 (2) Å
*c* = 20.0291 (2) Å
β = 99.982 (1)°
*V* = 7184.70 (13) Å^3^
*Z* = 16
Mo *K*α radiation
μ = 0.09 mm^−1^
*T* = 100 K
0.29 × 0.19 × 0.17 mm

#### Data collection

Bruker SMART APEXII CCD area-detector diffractometer
Absorption correction: multi-scan (**SADABS**; Bruker, 2005 ▶) *T*_min_ = 0.920, *T*_max_ = 0.984
67041 measured reflections
12647 independent reflections
8172 reflections with *I* > 2σ(*I*)
*R*_int_ = 0.069

#### Refinement

*R*[*F* ^2^ > 2σ(*F* ^2^)] = 0.048
*wR*(*F* ^2^) = 0.115
*S* = 1.03
12647 reflections
1075 parameters
H atoms treated by a mixture of independent and constrained refinement
Δρ_max_ = 0.24 e Å^−3^
Δρ_min_ = −0.22 e Å^−3^

### 

Data collection: *APEX2* (Bruker, 2005 ▶); cell refinement: *SAINT* (Bruker, 2005 ▶); data reduction: *SAINT*; program(s) used to solve structure: *SHELXTL* (Sheldrick, 2008 ▶); program(s) used to refine structure: *SHELXTL*; molecular graphics: *SHELXTL* software used to prepare material for publication: *SHELXTL* and *PLATON* (Spek, 2009 ▶).

## Supplementary Material

Crystal structure: contains datablocks global, I. DOI: 10.1107/S1600536809029833/ci2856sup1.cif

Structure factors: contains datablocks I. DOI: 10.1107/S1600536809029833/ci2856Isup2.hkl

Additional supplementary materials:  crystallographic information; 3D view; checkCIF report

## Figures and Tables

**Table 1:** Hydrogen-bond geometry (Å, °)

*D*—H⋯*A*	*D*—H	H⋯*A*	*D*⋯*A*	*D*—H⋯*A*
O2*A*—H2*OA*⋯N1*A*	0.92 (3)	1.86 (3)	2.686 (3)	149 (3)
O2*B*—H2*OB*⋯N1*B*	0.96 (3)	1.82 (3)	2.671 (2)	146 (2)
O2*C*—H2*OC*⋯N1*C*	0.99 (3)	1.72 (3)	2.634 (3)	151 (3)
O2*D*—H2*OD*⋯N1*D*	0.95 (3)	1.81 (3)	2.652 (3)	147 (3)
N2*A*—H2*NA*⋯O3*A*	0.92 (3)	1.86 (3)	2.631 (3)	140 (2)
N2*B*—H2*NB*⋯O3*B*	1.03 (3)	1.70 (3)	2.596 (2)	144 (3)
N2*C*—H2*NC*⋯O3*C*	0.95 (3)	1.83 (2)	2.620 (3)	139 (2)
N2*D*—H2*ND*⋯O3*D*	0.97 (3)	1.80 (3)	2.624 (2)	140 (2)
O1*C*—H1*OC*⋯O3*D*	0.88 (3)	1.91 (4)	2.734 (3)	155 (3)
O1*D*—H1*OD*⋯O3*C*	0.91 (3)	1.93 (3)	2.724 (2)	146 (3)
O4*C*—H4*OC*⋯O2*D*	0.88 (4)	2.48 (4)	3.079 (3)	126 (3)
O4*C*—H4*OC*⋯O3*D*	0.88 (4)	2.31 (4)	3.026 (2)	138 (3)
O4*D*—H4*OD*⋯O2*C*	0.85 (3)	2.34 (3)	2.942 (2)	129 (3)
O4*D*—H4*OD*⋯O3*C*	0.85 (3)	2.29 (3)	2.943 (3)	134 (3)
O1*A*—H1*OA*⋯O3*B*^i^	0.89 (3)	1.88 (3)	2.732 (2)	160 (3)
O1*B*—H1*OB*⋯O3*A*^ii^	0.85 (3)	2.01 (3)	2.809 (3)	157 (3)
O4*A*—H4*OA*⋯O2*B*^i^	0.81 (4)	2.25 (4)	2.929 (3)	142 (4)
O4*B*—H4*OB*⋯O2*A*^ii^	0.91 (4)	2.33 (3)	3.021 (3)	133 (3)
O4*B*—H4*OB*⋯O3*A*^ii^	0.91 (4)	2.53 (4)	3.231 (3)	134 (3)
C5*B*—H5*BA*⋯O1*D*^iii^	0.93	2.52	3.372 (3)	152
C7*B*—H7*BA*⋯O1*D*^iii^	0.93	2.57	3.416 (3)	151
C14*A*—H14*A*⋯O4*D*^iv^	0.93	2.36	3.281 (3)	170
C14*B*—H14*B*⋯O1*C*^iv^	0.93	2.40	3.260 (3)	154
C14*C*—H14*C*⋯O4*B*^v^	0.93	2.46	3.323 (3)	154
C10*D*—H10*D*⋯*Cg*1^vi^	0.93	2.97	3.623 (3)	128
C9*A*—H9*AA*⋯*Cg*2^vii^	0.93	2.97	3.716 (3)	138
C9*C*—H9*CA*⋯*Cg*3	0.93	2.65	3.480 (3)	148
C21*C*—H21*I*⋯*Cg*4^v^	0.96	2.95	3.696 (4)	136
C4*B*—H4*BA*⋯*Cg*5^viii^	0.93	2.83	3.573 (3)	138
C17*D*—H17*D*⋯*Cg*6^ix^	0.93	2.69	3.450 (3)	139

Symmetry codes: (i) 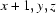; (ii) 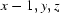; (iii) 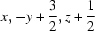; (iv) 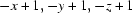; (v) 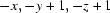; (vi) 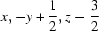; (vii) 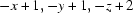; (viii) 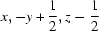; (ix) 
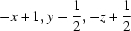
. *Cg*1, *Cg*2, *Cg*3, *Cg*4, *Cg*5 and *Cg*6 are the centroids of the C15*A*–C20*A*, C1*B*–C6*B*, C8*B*–C13*B*, C15*B*–C20*B*, C8*C*–C13*C* and C1*D*–C6*D* rings, respectively.

**Fig. 1.**
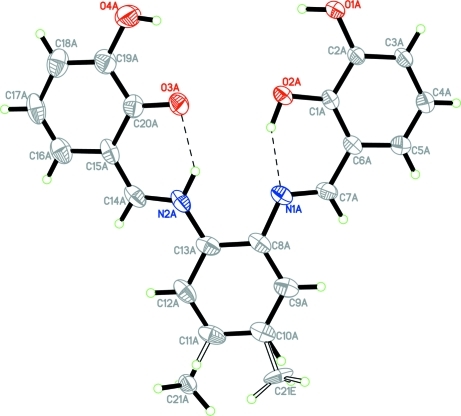
Molecule A of the title compound, with atom labels and 50% probability ellipsoids for non-H atoms. The other three independent molecules are labelled similarly. Intramolecular hydrogen bonds are shown as dashed lines. Both disorder components are shown.

**Fig. 2.**
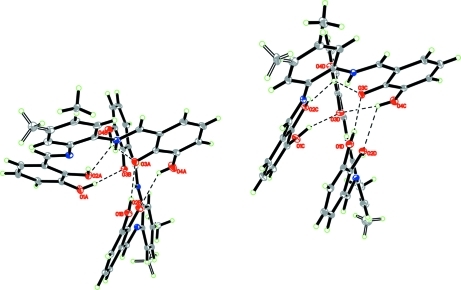
The asymmetric unit of the title compound, showing four independent molecules. Displacement ellipsoids are drawn at the 20% probability level. For clarity, only O and N atoms are labelled. Intermolecular hydrogen bonds are shown as dashed lines.

**Fig. 3.**
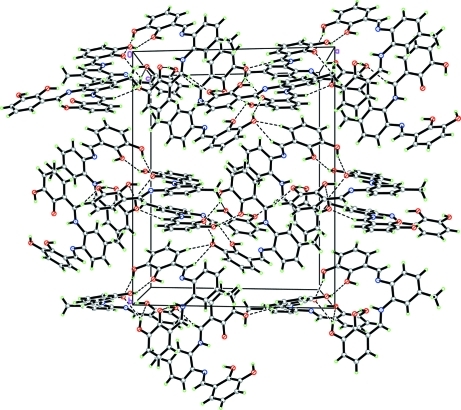
The crystal packing of the title compound, viewed down the c axis. Intermolecular hydrogen bonds are shown as dashed lines.

## supplementary crystallographic information

### Comment

Schiff bases have received much attention because of their potential applications with some of these compounds exhibiting various pharmacological activities, as noted by their anticancer (Dao *et al.*, 2000), anti-HIV (Sriram *et al.*, 2006), antibacterial and antifungal (Karthikeyan *et al.*, 2006) properties. In addition, some of them may be used as analytical reagents for the determination of trace elements (Eltayeb & Ahmed, 2005*a*,b). Previously, we have reported the crystal structures of 2,2'-[1,2-phenylenebis(nitrilomethylidyne)]bis(5-methylphenol) (Eltayeb *et al.*, 2007*a*) and 6,6'-dimethyl-2,2'-[1,2-phenylenebis(nitrilomethylidyne)]diphenol (Eltayeb *et al.*, 2007*b*). In this paper, we report the crystal structure of the title compound, obtained by the reaction of 4-methyl-1,2-phenylenediamine and 2,3-dihydroxybenzaldehyde.

The asymmetric unit of title compound consists of four crystallographically independent zwitterions *A*, *B*, *C* and *D* with almost similar conformations (Fig. 1 and Fig. 2). Each zwitterion is formed by the transfer one of the *ortho*-hydroxyl proton onto the adjacent imine unit resulting in the formation of iminium and hydroxyphenolate groups, with corresponding changes in the C20—O3, C14—N2 and C14—C15 bond lengths. These bonds are comparable to those in the related structure (Eltayeb *et al.*, 2008). The dihydroxyphenyl ring [C1–C6] and the hydroxyphenolate ring [C15–C20] in molecule *A* form dihedral angles of 17.36 (12) and 13.30 (12)°, respectively, with the benzene ring (C8–C13) whereas these angles for molecules *B*, *C* and *D* are 30.22 (11)/7.46 (11)°, 35.26 (12)/11.01 (12)° and 39.89 (12)/4.29 (12)°, respectively.

In each independent molecule, each intramolecular N—H···O and O—H···N hydrogen bonds (Table 1) generate an *S*(6) ring motif (Bernstein *et al.*, 1995). The four independent molecules are linked into two pairs, *A*/*B* and *C*/*D via* intermolecular O—H···O hydrogen bonds (Fig. 2 and Table 1). Intermolecular C—H···O interactions link these pairs of molecules into two-dimensional networks parallel to *ab* plane (Fig. 3). In addition, C—H···π (Table 1) and π–π interactions [*Cg*6···*Cg*8 = 3.5534 (14) Å, *Cg*3···*Cg*11^iv^ = 3.5153 (14) Å, *Cg*7···*Cg*9^iv^ = 3.7810 (15) Å, *Cg*1···*Cg*5^iv^ = 3.7581 (15) Å and *Cg*4···*Cg*10^iv^ = 3.7518 (15) Å, where *Cg*1, *Cg*3, *Cg*4, *Cg*5, *Cg*6, *Cg*7, *Cg*8, *Cg*9, *Cg*10 and *Cg*11 are centroids of C15A–C20A, C8B–C13B, C15B–C20B, C8C–C13C, C1D–C6D, C8A–C13A, C1C–C6C, C15C–C20C, C8D–C13D, C15D–C20D benzene rings, respectively] stabilize the crystal structure.

### Experimental

A solution of 4-methyl-1,2-phenylenediamine (0.244 g, 2 mmol) in ethanol (20 ml) was added into 2,3-dihydroxybenzaldehyde (0.552 g, 4 mmol). The mixture was refluxed with stirring for 30 min. The resultant red solution was filtered. Red crystals suitable for X-ray analysis were formed after a few days of slow evaporation of the solvent at room temperature.

### Refinement

All O and N bound H atoms were located in a difference Fourier map and refined freely. The rest of the H atoms were positioned geometrically and refined using a riding model, with C–H = 0.93 or 0.96 Å and *U*_iso_(H) = 1.2-1.5(methyl)*U*_eq_(C). The rotating group model was applied for the methyl groups. The methyl group in each independent molecule is disordered over two positions, with refined site-occupancies of 0.819 (5) and 0.181 (5) for molecule *A*, 0.912 (5) and 0.088 (5) for *B*, 0.734 (5) and 0.266 (5) for *C*, and 0.940 (6) and 0.060 (6) for *D*. Atoms C21F and C21H were refined isotropically.

### Crystal data

**Table:**

C_21_H_18_N_2_O_4_	*F*(000) = 3040
*M**_r_* = 362.37	*D*_x_ = 1.340 Mg m^−^^3^
Monoclinic, *P*2_1_/*c*	Mo *K*α radiation, λ = 0.71073 Å
Hall symbol: -P 2ybc	Cell parameters from 9276 reflections
*a* = 17.0634 (2) Å	θ = 2.2–29.6°
*b* = 21.3455 (2) Å	µ = 0.09 mm^−^^1^
*c* = 20.0291 (2) Å	*T* = 100 K
β = 99.982 (1)°	Block, red
*V* = 7184.70 (13) Å^3^	0.29 × 0.19 × 0.17 mm
*Z* = 16	

### Data collection

**Table:**

Bruker SMART APEXII CCD area-detector diffractometer	12647 independent reflections
Radiation source: fine-focus sealed tube	8172 reflections with *I* > 2σ(*I*)
graphite	*R*_int_ = 0.069
φ and ω scans	θ_max_ = 25.0°, θ_min_ = 2.1°
Absorption correction: multi-scan (*SADABS*; Bruker, 2005)	*h* = −20→20
*T*_min_ = 0.920, *T*_max_ = 0.984	*k* = −25→25
67041 measured reflections	*l* = −23→23

### Refinement

**Table:**

Refinement on *F*^2^	Primary atom site location: structure-invariant direct methods
Least-squares matrix: full	Secondary atom site location: difference Fourier map
*R*[*F*^2^ > 2σ(*F*^2^)] = 0.048	Hydrogen site location: inferred from neighbouring sites
*wR*(*F*^2^) = 0.115	H atoms treated by a mixture of independent and constrained refinement
*S* = 1.03	*w* = 1/[σ^2^(*F*_o_^2^) + (0.0429*P*)^2^ + 2.6321*P*] where *P* = (*F*_o_^2^ + 2*F*_c_^2^)/3
12647 reflections	(Δ/σ)_max_ = 0.001
1075 parameters	Δρ_max_ = 0.24 e Å^−^^3^
0 restraints	Δρ_min_ = −0.22 e Å^−^^3^

### Special details

**Table:**

Experimental. The crystal was placed in the cold stream of an Oxford Cyrosystems Cobra open-flow nitrogen cryostat (Cosier & Glazer, 1986) operating at 100.0 (1) K.
Geometry. All e.s.d.'s (except the e.s.d. in the dihedral angle between two l.s. planes) are estimated using the full covariance matrix. The cell e.s.d.'s are taken into account individually in the estimation of e.s.d.'s in distances, angles and torsion angles; correlations between e.s.d.'s in cell parameters are only used when they are defined by crystal symmetry. An approximate (isotropic) treatment of cell e.s.d.'s is used for estimating e.s.d.'s involving l.s. planes.
Refinement. Refinement of *F*^2^ against ALL reflections. The weighted *R*-factor *wR* and goodness of fit *S* are based on *F*^2^, conventional *R*-factors *R* are based on *F*, with *F* set to zero for negative *F*^2^. The threshold expression of *F*^2^ > σ(*F*^2^) is used only for calculating *R*-factors(gt) *etc*. and is not relevant to the choice of reflections for refinement. *R*-factors based on *F*^2^ are statistically about twice as large as those based on *F*, and *R*- factors based on ALL data will be even larger.

### Fractional atomic coordinates and isotropic or equivalent isotropic displacement parameters (Å^2^)

**Table:**

	*x*	*y*	*z*	*U*_iso_*/*U*_eq_	Occ. (<1)
O1A	1.19315 (10)	0.46141 (9)	0.86061 (9)	0.0364 (5)	
O2A	1.03690 (10)	0.47712 (8)	0.86453 (9)	0.0331 (4)	
O3A	0.91941 (9)	0.51191 (9)	0.75558 (9)	0.0397 (5)	
O4A	0.97187 (13)	0.53499 (12)	0.63763 (11)	0.0620 (7)	
N1A	0.91419 (11)	0.45158 (9)	0.92792 (10)	0.0303 (5)	
N2A	0.80118 (13)	0.48196 (10)	0.81813 (12)	0.0327 (5)	
C1A	1.08033 (13)	0.44352 (11)	0.91497 (12)	0.0259 (5)	
C2A	1.16102 (13)	0.43568 (12)	0.91227 (12)	0.0286 (6)	
C3A	1.20934 (14)	0.40307 (12)	0.96296 (12)	0.0348 (6)	
H3AA	1.2630	0.3980	0.9610	0.042*	
C4A	1.17861 (15)	0.37790 (14)	1.01666 (13)	0.0420 (7)	
H4AA	1.2116	0.3561	1.0506	0.050*	
C5A	1.09910 (14)	0.38517 (13)	1.01976 (13)	0.0391 (7)	
H5AA	1.0787	0.3685	1.0561	0.047*	
C6A	1.04886 (13)	0.41752 (11)	0.96865 (12)	0.0292 (6)	
C7A	0.96505 (14)	0.42262 (12)	0.97218 (13)	0.0325 (6)	
H7AA	0.9469	0.4039	1.0086	0.039*	
C8A	0.83324 (14)	0.45305 (11)	0.93647 (14)	0.0319 (6)	
C9A	0.80711 (15)	0.44014 (12)	0.99758 (14)	0.0368 (6)	
H9AA	0.8443	0.4312	1.0361	0.044*	
C10A	0.72750 (15)	0.44047 (12)	1.00179 (15)	0.0375 (7)	
H10A	0.7117	0.4310	1.0428	0.045*	0.819 (5)
C11A	0.67088 (15)	0.45459 (11)	0.94612 (15)	0.0374 (7)	
H11A	0.6171	0.4545	0.9492	0.045*	0.181 (5)
C12A	0.69529 (14)	0.46899 (11)	0.88544 (15)	0.0373 (7)	
H12A	0.6576	0.4795	0.8478	0.045*	
C13A	0.77596 (14)	0.46789 (11)	0.88016 (14)	0.0330 (6)	
C14A	0.75666 (14)	0.48370 (11)	0.75795 (14)	0.0338 (6)	
H14A	0.7029	0.4742	0.7545	0.041*	
C15A	0.78507 (14)	0.49897 (11)	0.69828 (13)	0.0323 (6)	
C16A	0.73153 (16)	0.50164 (12)	0.63598 (15)	0.0416 (7)	
H16A	0.6778	0.4934	0.6351	0.050*	
C17A	0.75728 (16)	0.51593 (13)	0.57788 (15)	0.0457 (7)	
H17A	0.7211	0.5182	0.5375	0.055*	
C18A	0.83918 (17)	0.52746 (13)	0.57792 (14)	0.0458 (7)	
H18A	0.8566	0.5369	0.5376	0.055*	
C19A	0.89239 (16)	0.52477 (13)	0.63700 (14)	0.0418 (7)	
C20A	0.86793 (15)	0.51179 (12)	0.70029 (13)	0.0351 (6)	
C21A	0.58355 (16)	0.45353 (14)	0.95249 (15)	0.0316 (9)	0.819 (5)
H21A	0.5788	0.4550	0.9995	0.047*	0.819 (5)
H21B	0.5594	0.4158	0.9325	0.047*	0.819 (5)
H21C	0.5572	0.4892	0.9295	0.047*	0.819 (5)
C21E	0.6917 (9)	0.4348 (8)	1.0509 (7)	0.045 (5)	0.181 (5)
H21M	0.7200	0.4571	1.0892	0.067*	0.181 (5)
H21N	0.6886	0.3913	1.0622	0.067*	0.181 (5)
H21O	0.6389	0.4516	1.0390	0.067*	0.181 (5)
O1B	−0.02790 (9)	0.62281 (9)	0.82457 (9)	0.0343 (4)	
O2B	0.08476 (9)	0.58736 (8)	0.75073 (8)	0.0323 (4)	
O3B	0.11961 (9)	0.45970 (8)	0.72811 (8)	0.0287 (4)	
O4B	0.00762 (10)	0.37802 (10)	0.75534 (9)	0.0380 (5)	
N1B	0.23622 (11)	0.59434 (9)	0.73336 (9)	0.0262 (5)	
N2B	0.24301 (11)	0.48183 (9)	0.67198 (10)	0.0253 (5)	
C1B	0.10696 (13)	0.62649 (11)	0.80378 (11)	0.0255 (5)	
C2B	0.04828 (13)	0.64443 (11)	0.84062 (12)	0.0269 (6)	
C3B	0.06718 (14)	0.68648 (11)	0.89352 (12)	0.0285 (6)	
H3BA	0.0278	0.6996	0.9172	0.034*	
C4B	0.14418 (14)	0.70940 (11)	0.91184 (12)	0.0287 (6)	
H4BA	0.1560	0.7376	0.9476	0.034*	
C5B	0.20283 (14)	0.69050 (11)	0.87721 (11)	0.0278 (6)	
H5BA	0.2545	0.7054	0.8901	0.033*	
C6B	0.18514 (13)	0.64872 (11)	0.82217 (11)	0.0251 (5)	
C7B	0.24725 (13)	0.63130 (11)	0.78509 (12)	0.0272 (6)	
H7BA	0.2979	0.6476	0.7993	0.033*	
C8B	0.30034 (13)	0.58367 (11)	0.69796 (11)	0.0254 (5)	
C9B	0.35955 (13)	0.62756 (12)	0.69341 (11)	0.0294 (6)	
H9BA	0.3582	0.6664	0.7142	0.035*	
C10B	0.42014 (14)	0.61392 (13)	0.65826 (12)	0.0322 (6)	
H10B	0.4600	0.6434	0.6568	0.039*	0.912 (5)
C11B	0.42286 (13)	0.55688 (13)	0.62487 (12)	0.0326 (6)	
H11B	0.4636	0.5483	0.6009	0.039*	0.088 (5)
C12B	0.36306 (13)	0.51301 (12)	0.62826 (12)	0.0298 (6)	
H12B	0.3636	0.4748	0.6059	0.036*	
C13B	0.30267 (13)	0.52601 (12)	0.66482 (11)	0.0265 (6)	
C14B	0.23733 (13)	0.42342 (12)	0.65070 (12)	0.0278 (6)	
H14B	0.2745	0.4085	0.6257	0.033*	
C15B	0.17722 (13)	0.38248 (11)	0.66421 (12)	0.0266 (6)	
C16B	0.17535 (14)	0.31955 (12)	0.64055 (13)	0.0355 (6)	
H16B	0.2126	0.3062	0.6148	0.043*	
C17B	0.11951 (15)	0.27888 (13)	0.65529 (13)	0.0392 (7)	
H17B	0.1188	0.2377	0.6399	0.047*	
C18B	0.06269 (14)	0.29908 (13)	0.69389 (13)	0.0361 (6)	
H18B	0.0244	0.2711	0.7035	0.043*	
C19B	0.06291 (13)	0.35899 (12)	0.71741 (12)	0.0298 (6)	
C20B	0.12030 (13)	0.40368 (12)	0.70386 (12)	0.0270 (6)	
C21B	0.48833 (16)	0.54276 (13)	0.58557 (15)	0.0375 (9)	0.912 (5)
H21D	0.5302	0.5730	0.5967	0.056*	0.912 (5)
H21E	0.4676	0.5447	0.5379	0.056*	0.912 (5)
H21F	0.5090	0.5016	0.5970	0.056*	0.912 (5)
C21F	0.5082 (15)	0.6287 (13)	0.6610 (14)	0.033 (8)*	0.088 (5)
H21S	0.5161	0.6732	0.6643	0.049*	0.088 (5)
H21T	0.5248	0.6136	0.6205	0.049*	0.088 (5)
H21U	0.5389	0.6087	0.6998	0.049*	0.088 (5)
O1C	0.61613 (10)	0.65594 (9)	0.39257 (10)	0.0369 (5)	
O2C	0.46109 (10)	0.63085 (8)	0.39791 (8)	0.0291 (4)	
O3C	0.34742 (9)	0.67350 (8)	0.28067 (8)	0.0296 (4)	
O4C	0.40206 (10)	0.69348 (9)	0.16221 (10)	0.0411 (5)	
N1C	0.33732 (11)	0.65233 (9)	0.45702 (9)	0.0263 (5)	
N2C	0.22571 (12)	0.65583 (9)	0.34223 (10)	0.0251 (5)	
C1C	0.50298 (13)	0.66524 (11)	0.44890 (11)	0.0255 (5)	
C2C	0.58275 (14)	0.67692 (11)	0.44600 (12)	0.0288 (6)	
C3C	0.62882 (14)	0.70965 (13)	0.49781 (13)	0.0369 (6)	
H3CA	0.6825	0.7163	0.4967	0.044*	
C4C	0.59594 (15)	0.73281 (13)	0.55160 (13)	0.0397 (7)	
H4CA	0.6278	0.7544	0.5865	0.048*	
C5C	0.51658 (14)	0.72393 (12)	0.55351 (12)	0.0323 (6)	
H5CA	0.4946	0.7406	0.5890	0.039*	
C6C	0.46872 (13)	0.68982 (11)	0.50200 (11)	0.0263 (5)	
C7C	0.38493 (14)	0.68150 (11)	0.50396 (12)	0.0269 (6)	
H7CA	0.3646	0.6978	0.5405	0.032*	
C8C	0.25654 (13)	0.64372 (11)	0.46340 (12)	0.0265 (6)	
C9C	0.23143 (15)	0.63346 (12)	0.52487 (13)	0.0348 (6)	
H9CA	0.2686	0.6342	0.5648	0.042*	
C10C	0.15256 (16)	0.62218 (13)	0.52787 (14)	0.0414 (7)	
H10C	0.1370	0.6163	0.5697	0.050*	0.734 (5)
C11C	0.09631 (15)	0.61948 (13)	0.46909 (14)	0.0391 (7)	
H11C	0.0433	0.6110	0.4711	0.047*	0.266 (5)
C12C	0.12025 (14)	0.62955 (12)	0.40712 (13)	0.0335 (6)	
H12C	0.0830	0.6278	0.3674	0.040*	
C13C	0.19956 (14)	0.64221 (11)	0.40397 (12)	0.0265 (6)	
C14C	0.18110 (14)	0.66724 (11)	0.28360 (12)	0.0280 (6)	
H14C	0.1261	0.6664	0.2809	0.034*	
C15C	0.21183 (13)	0.68077 (11)	0.22438 (12)	0.0266 (6)	
C16C	0.15820 (15)	0.69103 (12)	0.16293 (13)	0.0372 (7)	
H16C	0.1036	0.6905	0.1626	0.045*	
C17C	0.18628 (16)	0.70164 (13)	0.10459 (13)	0.0399 (7)	
H17C	0.1509	0.7086	0.0644	0.048*	
C18C	0.26869 (15)	0.70220 (12)	0.10455 (13)	0.0346 (6)	
H18C	0.2871	0.7088	0.0641	0.041*	
C19C	0.32201 (14)	0.69329 (11)	0.16285 (12)	0.0297 (6)	
C20C	0.29569 (14)	0.68228 (11)	0.22574 (12)	0.0262 (5)	
C21C	0.01231 (19)	0.6010 (2)	0.47405 (18)	0.0451 (12)	0.734 (5)
H21G	0.0080	0.5950	0.5208	0.068*	0.734 (5)
H21H	−0.0010	0.5627	0.4495	0.068*	0.734 (5)
H21I	−0.0236	0.6335	0.4550	0.068*	0.734 (5)
C21G	0.1144 (6)	0.6217 (6)	0.5868 (5)	0.047 (3)	0.266 (5)
H21P	0.1433	0.6483	0.6212	0.070*	0.266 (5)
H21Q	0.1137	0.5798	0.6039	0.070*	0.266 (5)
H21R	0.0608	0.6368	0.5746	0.070*	0.266 (5)
O1D	0.39194 (9)	0.77129 (8)	0.36623 (10)	0.0343 (4)	
O2D	0.49820 (10)	0.76787 (8)	0.28054 (9)	0.0335 (4)	
O3D	0.54298 (9)	0.64355 (8)	0.26057 (8)	0.0321 (4)	
O4D	0.43246 (10)	0.55434 (9)	0.27391 (9)	0.0377 (5)	
N1D	0.64614 (11)	0.79038 (9)	0.26308 (10)	0.0306 (5)	
N2D	0.66987 (11)	0.67631 (9)	0.21144 (10)	0.0253 (5)	
C1D	0.51759 (14)	0.80222 (11)	0.33813 (12)	0.0284 (6)	
C2D	0.46195 (14)	0.80328 (11)	0.38153 (13)	0.0304 (6)	
C3D	0.47734 (15)	0.83798 (12)	0.44060 (13)	0.0355 (6)	
H3DA	0.4406	0.8385	0.4699	0.043*	
C4D	0.54770 (15)	0.87227 (12)	0.45641 (13)	0.0379 (6)	
H4DA	0.5570	0.8966	0.4955	0.045*	
C5D	0.60308 (15)	0.87034 (12)	0.41466 (13)	0.0357 (6)	
H5DA	0.6500	0.8931	0.4258	0.043*	
C6D	0.58983 (14)	0.83434 (11)	0.35500 (13)	0.0307 (6)	
C7D	0.65207 (14)	0.82724 (12)	0.31460 (13)	0.0320 (6)	
H7DA	0.6985	0.8504	0.3265	0.038*	
C8D	0.71118 (14)	0.78384 (12)	0.22887 (12)	0.0304 (6)	
C9D	0.76177 (15)	0.83227 (13)	0.21815 (13)	0.0400 (7)	
H9DA	0.7533	0.8722	0.2342	0.048*	
C10D	0.82403 (17)	0.82238 (14)	0.18438 (14)	0.0463 (7)	
H10D	0.8572	0.8557	0.1782	0.056*	0.940 (6)
C11D	0.83857 (15)	0.76391 (14)	0.15938 (13)	0.0398 (7)	
H11D	0.8812	0.7576	0.1369	0.048*	0.060 (6)
C12D	0.78779 (14)	0.71468 (13)	0.16874 (12)	0.0341 (6)	
H12D	0.7964	0.6750	0.1523	0.041*	
C13D	0.72434 (13)	0.72481 (12)	0.20252 (12)	0.0273 (6)	
C14D	0.66857 (13)	0.61896 (12)	0.18750 (12)	0.0283 (6)	
H14D	0.7082	0.6074	0.1633	0.034*	
C15D	0.61139 (13)	0.57423 (11)	0.19609 (12)	0.0268 (6)	
C16D	0.61550 (14)	0.51327 (12)	0.16815 (13)	0.0354 (6)	
H16D	0.6558	0.5037	0.1439	0.042*	
C17D	0.56089 (14)	0.46882 (12)	0.17668 (13)	0.0360 (6)	
H17D	0.5642	0.4290	0.1586	0.043*	
C18D	0.49948 (14)	0.48311 (12)	0.21279 (12)	0.0303 (6)	
H18D	0.4626	0.4524	0.2184	0.036*	
C19D	0.49304 (13)	0.54098 (11)	0.23962 (11)	0.0260 (6)	
C20D	0.54951 (13)	0.58924 (11)	0.23323 (11)	0.0259 (6)	
C21D	0.90795 (16)	0.75283 (15)	0.12341 (16)	0.0490 (10)	0.940 (6)
H21J	0.9432	0.7882	0.1302	0.074*	0.940 (6)
H21K	0.8888	0.7474	0.0758	0.074*	0.940 (6)
H21L	0.9360	0.7158	0.1413	0.074*	0.940 (6)
C21H	0.900 (4)	0.848 (3)	0.164 (3)	0.08 (2)*	0.060 (6)
H21V	0.9173	0.8842	0.1913	0.127*	0.060 (6)
H21W	0.8895	0.8596	0.1172	0.127*	0.060 (6)
H21X	0.9405	0.8165	0.1714	0.127*	0.060 (6)
H1OA	1.161 (2)	0.4665 (16)	0.8211 (17)	0.085 (12)*	
H1OB	−0.0314 (16)	0.5904 (14)	0.7999 (14)	0.050 (10)*	
H1OC	0.581 (2)	0.6478 (17)	0.3561 (18)	0.094 (14)*	
H1OD	0.396 (2)	0.7436 (16)	0.3327 (17)	0.085 (12)*	
H2OA	0.9859 (19)	0.4770 (15)	0.8730 (16)	0.081 (11)*	
H2OB	0.1312 (17)	0.5778 (13)	0.7319 (14)	0.061 (9)*	
H2OC	0.4060 (18)	0.6322 (14)	0.4069 (15)	0.072 (10)*	
H2OD	0.5446 (18)	0.7653 (14)	0.2607 (15)	0.065 (10)*	
H4OA	0.997 (3)	0.533 (2)	0.676 (2)	0.130 (19)*	
H4OB	0.014 (2)	0.4197 (17)	0.7643 (17)	0.088 (13)*	
H4OC	0.426 (2)	0.6875 (19)	0.2045 (19)	0.110 (15)*	
H4OD	0.436 (2)	0.5922 (16)	0.2872 (17)	0.082 (13)*	
H2NA	0.8542 (18)	0.4897 (14)	0.8169 (14)	0.062 (9)*	
H2NB	0.1992 (18)	0.4912 (14)	0.6998 (14)	0.072 (10)*	
H2NC	0.2810 (15)	0.6585 (11)	0.3414 (12)	0.040 (7)*	
H2ND	0.6257 (16)	0.6842 (12)	0.2352 (13)	0.051 (8)*	

### Atomic displacement parameters (Å^2^)

**Table:**

	*U*^11^	*U*^22^	*U*^33^	*U*^12^	*U*^13^	*U*^23^
O1A	0.0209 (9)	0.0591 (13)	0.0297 (11)	−0.0017 (9)	0.0056 (9)	0.0028 (9)
O2A	0.0201 (9)	0.0424 (11)	0.0371 (10)	0.0054 (8)	0.0054 (8)	0.0064 (9)
O3A	0.0271 (9)	0.0517 (12)	0.0368 (11)	−0.0115 (9)	−0.0041 (9)	0.0051 (9)
O4A	0.0430 (13)	0.104 (2)	0.0374 (13)	−0.0274 (12)	0.0013 (11)	0.0070 (13)
N1A	0.0220 (11)	0.0296 (12)	0.0395 (13)	−0.0022 (9)	0.0060 (10)	−0.0041 (10)
N2A	0.0201 (11)	0.0263 (12)	0.0493 (15)	−0.0004 (9)	−0.0012 (11)	0.0061 (10)
C1A	0.0212 (12)	0.0264 (14)	0.0287 (14)	0.0022 (10)	0.0000 (11)	−0.0029 (11)
C2A	0.0210 (12)	0.0400 (16)	0.0247 (13)	0.0009 (11)	0.0033 (11)	−0.0030 (12)
C3A	0.0195 (12)	0.0508 (18)	0.0330 (15)	0.0020 (12)	0.0015 (12)	−0.0022 (13)
C4A	0.0285 (14)	0.060 (2)	0.0360 (16)	0.0028 (13)	0.0011 (12)	0.0113 (14)
C5A	0.0296 (14)	0.0522 (19)	0.0358 (16)	−0.0038 (13)	0.0067 (13)	0.0089 (14)
C6A	0.0211 (12)	0.0323 (15)	0.0339 (15)	0.0004 (11)	0.0034 (11)	−0.0005 (12)
C7A	0.0240 (13)	0.0358 (16)	0.0393 (16)	0.0007 (12)	0.0101 (12)	0.0021 (13)
C8A	0.0213 (13)	0.0260 (15)	0.0487 (17)	0.0010 (11)	0.0068 (13)	−0.0011 (12)
C9A	0.0314 (14)	0.0347 (16)	0.0466 (17)	0.0006 (12)	0.0132 (13)	−0.0049 (13)
C10A	0.0285 (14)	0.0337 (16)	0.0525 (18)	−0.0009 (12)	0.0128 (14)	−0.0038 (14)
C11A	0.0291 (14)	0.0231 (15)	0.064 (2)	−0.0011 (11)	0.0198 (15)	0.0003 (13)
C12A	0.0239 (13)	0.0250 (15)	0.0611 (19)	0.0034 (11)	0.0021 (13)	0.0035 (13)
C13A	0.0278 (14)	0.0209 (14)	0.0528 (17)	−0.0013 (11)	0.0140 (13)	0.0039 (12)
C14A	0.0216 (13)	0.0214 (14)	0.0567 (19)	−0.0001 (11)	0.0022 (14)	0.0020 (13)
C15A	0.0264 (13)	0.0189 (14)	0.0465 (17)	−0.0007 (11)	−0.0081 (13)	−0.0033 (12)
C16A	0.0341 (15)	0.0325 (17)	0.0541 (19)	−0.0015 (12)	−0.0038 (14)	−0.0025 (14)
C17A	0.0420 (17)	0.0375 (17)	0.0480 (19)	0.0023 (14)	−0.0187 (15)	−0.0061 (14)
C18A	0.0564 (19)	0.0385 (18)	0.0376 (17)	−0.0058 (14)	−0.0060 (15)	−0.0007 (13)
C19A	0.0370 (16)	0.0421 (18)	0.0427 (17)	−0.0107 (13)	−0.0032 (14)	−0.0004 (14)
C20A	0.0355 (15)	0.0308 (16)	0.0364 (16)	−0.0059 (12)	−0.0007 (13)	0.0021 (12)
C21A	0.0231 (16)	0.035 (2)	0.0370 (19)	0.0034 (14)	0.0049 (14)	0.0114 (15)
C21E	0.055 (10)	0.054 (11)	0.034 (9)	−0.007 (8)	0.032 (8)	−0.009 (8)
O1B	0.0224 (9)	0.0408 (12)	0.0392 (11)	−0.0016 (8)	0.0038 (8)	−0.0051 (10)
O2B	0.0224 (9)	0.0391 (11)	0.0353 (10)	−0.0022 (8)	0.0045 (8)	−0.0096 (9)
O3B	0.0265 (9)	0.0287 (10)	0.0308 (9)	−0.0024 (7)	0.0046 (8)	−0.0038 (8)
O4B	0.0315 (10)	0.0412 (12)	0.0421 (11)	−0.0112 (9)	0.0092 (9)	−0.0074 (10)
N1B	0.0233 (10)	0.0295 (12)	0.0257 (11)	−0.0024 (9)	0.0040 (9)	0.0008 (10)
N2B	0.0213 (10)	0.0284 (13)	0.0249 (11)	−0.0006 (9)	0.0005 (9)	−0.0005 (9)
C1B	0.0281 (13)	0.0237 (14)	0.0234 (13)	0.0000 (11)	0.0005 (11)	0.0010 (11)
C2B	0.0226 (13)	0.0274 (14)	0.0296 (14)	0.0017 (11)	0.0014 (11)	0.0069 (11)
C3B	0.0295 (13)	0.0294 (15)	0.0272 (14)	0.0050 (11)	0.0064 (11)	0.0059 (12)
C4B	0.0366 (14)	0.0235 (14)	0.0253 (13)	−0.0002 (11)	0.0040 (12)	0.0020 (11)
C5B	0.0266 (13)	0.0289 (15)	0.0264 (13)	−0.0038 (11)	0.0001 (11)	0.0028 (11)
C6B	0.0264 (13)	0.0235 (14)	0.0255 (13)	−0.0002 (10)	0.0047 (11)	0.0043 (11)
C7B	0.0224 (12)	0.0306 (15)	0.0270 (14)	−0.0054 (11)	0.0001 (11)	0.0039 (12)
C8B	0.0194 (12)	0.0324 (15)	0.0231 (13)	−0.0010 (11)	0.0006 (10)	0.0022 (11)
C9B	0.0299 (13)	0.0344 (15)	0.0228 (13)	−0.0064 (11)	0.0018 (11)	0.0021 (11)
C10B	0.0255 (13)	0.0451 (17)	0.0251 (13)	−0.0058 (12)	0.0022 (11)	0.0060 (13)
C11B	0.0219 (13)	0.0464 (17)	0.0298 (14)	0.0047 (12)	0.0056 (11)	0.0133 (13)
C12B	0.0266 (13)	0.0358 (16)	0.0261 (14)	0.0054 (11)	0.0022 (11)	0.0048 (11)
C13B	0.0198 (12)	0.0369 (16)	0.0218 (13)	−0.0023 (11)	0.0011 (10)	0.0070 (11)
C14B	0.0211 (12)	0.0366 (16)	0.0238 (13)	0.0049 (11)	−0.0011 (11)	−0.0008 (12)
C15B	0.0198 (12)	0.0281 (15)	0.0287 (14)	0.0012 (10)	−0.0045 (11)	−0.0007 (11)
C16B	0.0259 (13)	0.0373 (17)	0.0408 (16)	0.0034 (12)	−0.0010 (12)	−0.0083 (13)
C17B	0.0347 (15)	0.0315 (16)	0.0462 (17)	−0.0004 (12)	−0.0077 (13)	−0.0083 (13)
C18B	0.0281 (14)	0.0375 (17)	0.0395 (16)	−0.0079 (12)	−0.0028 (12)	−0.0031 (13)
C19B	0.0219 (13)	0.0362 (16)	0.0285 (14)	−0.0037 (11)	−0.0033 (11)	0.0007 (12)
C20B	0.0223 (12)	0.0302 (15)	0.0256 (13)	−0.0011 (11)	−0.0037 (11)	−0.0008 (11)
C21B	0.0323 (16)	0.0345 (18)	0.0495 (19)	0.0055 (13)	0.0175 (15)	0.0062 (14)
O1C	0.0245 (10)	0.0464 (12)	0.0390 (11)	0.0054 (8)	0.0035 (9)	−0.0122 (9)
O2C	0.0237 (9)	0.0327 (10)	0.0288 (10)	0.0025 (8)	−0.0011 (8)	−0.0074 (8)
O3C	0.0229 (9)	0.0368 (10)	0.0267 (9)	−0.0018 (7)	−0.0021 (8)	0.0042 (8)
O4C	0.0310 (10)	0.0567 (13)	0.0360 (12)	−0.0101 (9)	0.0069 (9)	0.0044 (10)
N1C	0.0255 (11)	0.0289 (12)	0.0237 (11)	0.0034 (9)	0.0020 (9)	0.0028 (9)
N2C	0.0236 (11)	0.0236 (12)	0.0271 (12)	0.0001 (9)	0.0014 (10)	0.0010 (9)
C1C	0.0266 (13)	0.0252 (14)	0.0219 (13)	0.0038 (11)	−0.0038 (11)	−0.0002 (11)
C2C	0.0263 (13)	0.0310 (15)	0.0272 (14)	0.0065 (11)	−0.0003 (11)	−0.0008 (12)
C3C	0.0225 (13)	0.0478 (18)	0.0374 (16)	0.0005 (12)	−0.0035 (12)	−0.0044 (14)
C4C	0.0346 (15)	0.0492 (18)	0.0309 (15)	−0.0034 (13)	−0.0068 (13)	−0.0050 (13)
C5C	0.0346 (15)	0.0368 (16)	0.0238 (14)	0.0034 (12)	0.0006 (12)	−0.0023 (12)
C6C	0.0280 (13)	0.0260 (14)	0.0233 (13)	0.0052 (11)	−0.0001 (11)	0.0025 (11)
C7C	0.0318 (14)	0.0259 (14)	0.0227 (13)	0.0040 (11)	0.0036 (11)	0.0034 (11)
C8C	0.0256 (13)	0.0244 (14)	0.0294 (14)	0.0017 (10)	0.0049 (11)	0.0013 (11)
C9C	0.0386 (15)	0.0385 (16)	0.0271 (14)	−0.0011 (12)	0.0047 (12)	0.0030 (12)
C10C	0.0397 (16)	0.0514 (19)	0.0362 (16)	−0.0084 (14)	0.0152 (14)	0.0001 (14)
C11C	0.0289 (14)	0.0431 (17)	0.0467 (17)	−0.0053 (12)	0.0106 (13)	−0.0028 (14)
C12C	0.0244 (13)	0.0367 (16)	0.0377 (15)	−0.0036 (11)	0.0009 (12)	0.0002 (13)
C13C	0.0280 (13)	0.0220 (14)	0.0302 (14)	0.0027 (10)	0.0068 (11)	0.0019 (11)
C14C	0.0253 (13)	0.0261 (14)	0.0304 (14)	0.0000 (11)	−0.0014 (11)	−0.0016 (11)
C15C	0.0260 (13)	0.0246 (14)	0.0278 (14)	−0.0014 (10)	0.0007 (11)	0.0011 (11)
C16C	0.0274 (14)	0.0459 (18)	0.0347 (16)	0.0000 (12)	−0.0046 (12)	0.0028 (13)
C17C	0.0402 (16)	0.0441 (18)	0.0313 (15)	−0.0036 (13)	−0.0055 (13)	0.0060 (13)
C18C	0.0446 (16)	0.0317 (16)	0.0268 (14)	−0.0077 (12)	0.0044 (13)	0.0042 (12)
C19C	0.0316 (14)	0.0268 (15)	0.0299 (14)	−0.0058 (11)	0.0037 (12)	0.0002 (11)
C20C	0.0297 (13)	0.0181 (13)	0.0288 (14)	−0.0019 (10)	−0.0002 (11)	0.0006 (11)
C21C	0.027 (2)	0.069 (3)	0.038 (2)	−0.0055 (19)	0.0046 (17)	0.009 (2)
C21G	0.036 (6)	0.076 (9)	0.027 (6)	−0.005 (6)	0.004 (5)	0.019 (6)
O1D	0.0259 (9)	0.0301 (11)	0.0460 (12)	0.0031 (8)	0.0037 (9)	0.0005 (9)
O2D	0.0280 (10)	0.0285 (10)	0.0428 (11)	−0.0005 (8)	0.0026 (9)	−0.0054 (8)
O3D	0.0330 (9)	0.0264 (10)	0.0377 (10)	−0.0069 (8)	0.0088 (8)	−0.0068 (8)
O4D	0.0372 (11)	0.0342 (12)	0.0443 (12)	−0.0134 (9)	0.0142 (9)	−0.0092 (9)
N1D	0.0303 (11)	0.0249 (12)	0.0353 (12)	−0.0005 (9)	0.0023 (10)	0.0026 (10)
N2D	0.0194 (10)	0.0268 (12)	0.0281 (11)	−0.0023 (9)	−0.0001 (9)	0.0025 (10)
C1D	0.0295 (14)	0.0194 (14)	0.0334 (15)	0.0044 (11)	−0.0026 (12)	0.0021 (11)
C2D	0.0280 (14)	0.0214 (14)	0.0402 (16)	0.0075 (11)	0.0012 (12)	0.0050 (12)
C3D	0.0332 (15)	0.0326 (16)	0.0401 (16)	0.0090 (12)	0.0045 (13)	0.0037 (13)
C4D	0.0379 (15)	0.0357 (16)	0.0376 (16)	0.0047 (13)	0.0000 (13)	−0.0030 (13)
C5D	0.0297 (14)	0.0316 (16)	0.0422 (16)	0.0020 (12)	−0.0041 (13)	−0.0013 (13)
C6D	0.0291 (14)	0.0248 (14)	0.0359 (15)	0.0052 (11)	−0.0009 (12)	0.0028 (12)
C7D	0.0254 (13)	0.0279 (15)	0.0390 (16)	−0.0023 (11)	−0.0046 (12)	0.0068 (13)
C8D	0.0268 (13)	0.0299 (15)	0.0325 (14)	−0.0041 (11)	−0.0002 (12)	0.0051 (12)
C9D	0.0459 (16)	0.0308 (16)	0.0434 (17)	−0.0101 (13)	0.0081 (14)	0.0021 (13)
C10D	0.0480 (17)	0.0411 (19)	0.0511 (18)	−0.0148 (14)	0.0119 (15)	0.0058 (15)
C11D	0.0280 (14)	0.0517 (19)	0.0397 (16)	−0.0071 (13)	0.0061 (13)	0.0080 (14)
C12D	0.0263 (13)	0.0379 (16)	0.0361 (15)	−0.0034 (12)	−0.0001 (12)	0.0007 (12)
C13D	0.0177 (12)	0.0352 (16)	0.0258 (13)	−0.0049 (11)	−0.0047 (10)	0.0057 (11)
C14D	0.0198 (12)	0.0322 (16)	0.0313 (14)	0.0038 (11)	0.0004 (11)	−0.0012 (12)
C15D	0.0186 (12)	0.0279 (15)	0.0314 (14)	0.0004 (10)	−0.0024 (11)	0.0006 (11)
C16D	0.0247 (13)	0.0331 (16)	0.0475 (17)	0.0048 (12)	0.0040 (12)	−0.0042 (13)
C17D	0.0321 (14)	0.0210 (14)	0.0506 (17)	0.0035 (12)	−0.0049 (13)	−0.0070 (13)
C18D	0.0249 (13)	0.0267 (15)	0.0352 (15)	−0.0033 (11)	−0.0062 (11)	0.0011 (12)
C19D	0.0229 (12)	0.0288 (15)	0.0247 (13)	−0.0034 (11)	−0.0006 (11)	0.0020 (11)
C20D	0.0240 (13)	0.0251 (15)	0.0255 (13)	0.0002 (11)	−0.0039 (11)	0.0016 (11)
C21D	0.0363 (17)	0.056 (2)	0.059 (2)	−0.0139 (15)	0.0185 (16)	−0.0038 (17)

### Geometric parameters (Å, °)

**Table:**

O1A—C2A	1.367 (3)	O1C—C2C	1.372 (3)
O1A—H1OA	0.89 (3)	O1C—H1OC	0.87 (4)
O2A—C1A	1.351 (3)	O2C—C1C	1.356 (3)
O2A—H2OA	0.91 (3)	O2C—H2OC	0.99 (3)
O3A—C20A	1.289 (3)	O3C—C20C	1.299 (3)
O4A—C19A	1.372 (3)	O4C—C19C	1.368 (3)
O4A—H4OA	0.81 (4)	O4C—H4OC	0.89 (4)
N1A—C7A	1.286 (3)	N1C—C7C	1.291 (3)
N1A—C8A	1.421 (3)	N1C—C8C	1.418 (3)
N2A—C14A	1.309 (3)	N2C—C14C	1.307 (3)
N2A—C13A	1.416 (3)	N2C—C13C	1.416 (3)
N2A—H2NA	0.92 (3)	N2C—H2NC	0.95 (2)
C1A—C2A	1.397 (3)	C1C—C2C	1.395 (3)
C1A—C6A	1.397 (3)	C1C—C6C	1.401 (3)
C2A—C3A	1.380 (3)	C2C—C3C	1.378 (3)
C3A—C4A	1.384 (3)	C3C—C4C	1.389 (3)
C3A—H3AA	0.93	C3C—H3CA	0.93
C4A—C5A	1.378 (3)	C4C—C5C	1.375 (3)
C4A—H4AA	0.93	C4C—H4CA	0.93
C5A—C6A	1.398 (3)	C5C—C6C	1.403 (3)
C5A—H5AA	0.93	C5C—H5CA	0.93
C6A—C7A	1.448 (3)	C6C—C7C	1.448 (3)
C7A—H7AA	0.93	C7C—H7CA	0.93
C8A—C13A	1.395 (3)	C8C—C9C	1.389 (3)
C8A—C9A	1.401 (3)	C8C—C13C	1.401 (3)
C9A—C10A	1.376 (3)	C9C—C10C	1.379 (3)
C9A—H9AA	0.93	C9C—H9CA	0.93
C10A—C21E	1.251 (12)	C10C—C11C	1.385 (4)
C10A—C11A	1.376 (4)	C10C—C21G	1.445 (9)
C10A—H10A	0.93	C10C—H10C	0.93
C11A—C12A	1.386 (3)	C11C—C12C	1.389 (3)
C11A—C21A	1.518 (3)	C11C—C21C	1.506 (4)
C11A—H11A	0.93	C11C—H11C	0.93
C12A—C13A	1.399 (3)	C12C—C13C	1.392 (3)
C12A—H12A	0.93	C12C—H12C	0.93
C14A—C15A	1.404 (3)	C14C—C15C	1.407 (3)
C14A—H14A	0.93	C14C—H14C	0.93
C15A—C16A	1.414 (4)	C15C—C16C	1.418 (3)
C15A—C20A	1.434 (3)	C15C—C20C	1.427 (3)
C16A—C17A	1.348 (4)	C16C—C17C	1.357 (3)
C16A—H16A	0.93	C16C—H16C	0.93
C17A—C18A	1.419 (4)	C17C—C18C	1.406 (3)
C17A—H17A	0.93	C17C—H17C	0.93
C18A—C19A	1.362 (4)	C18C—C19C	1.364 (3)
C18A—H18A	0.93	C18C—H18C	0.93
C19A—C20A	1.429 (3)	C19C—C20C	1.428 (3)
C21A—H21A	0.96	C21C—H21G	0.96
C21A—H21B	0.96	C21C—H21H	0.96
C21A—H21C	0.96	C21C—H21I	0.96
C21E—H21M	0.96	C21G—H21P	0.96
C21E—H21N	0.96	C21G—H21Q	0.96
C21E—H21O	0.96	C21G—H21R	0.96
O1B—C2B	1.364 (3)	O1D—C2D	1.364 (3)
O1B—H1OB	0.85 (3)	O1D—H1OD	0.91 (3)
O2B—C1B	1.353 (3)	O2D—C1D	1.358 (3)
O2B—H2OB	0.96 (3)	O2D—H2OD	0.95 (3)
O3B—C20B	1.292 (3)	O3D—C20D	1.295 (3)
O4B—C19B	1.371 (3)	O4D—C19D	1.367 (3)
O4B—H4OB	0.91 (4)	O4D—H4OD	0.85 (3)
N1B—C7B	1.290 (3)	N1D—C7D	1.288 (3)
N1B—C8B	1.422 (3)	N1D—C8D	1.409 (3)
N2B—C14B	1.316 (3)	N2D—C14D	1.313 (3)
N2B—C13B	1.413 (3)	N2D—C13D	1.423 (3)
N2B—H2NB	1.03 (3)	N2D—H2ND	0.97 (3)
C1B—C2B	1.396 (3)	C1D—C2D	1.394 (3)
C1B—C6B	1.404 (3)	C1D—C6D	1.400 (3)
C2B—C3B	1.383 (3)	C2D—C3D	1.382 (3)
C3B—C4B	1.390 (3)	C3D—C4D	1.395 (3)
C3B—H3BA	0.93	C3D—H3DA	0.93
C4B—C5B	1.373 (3)	C4D—C5D	1.367 (3)
C4B—H4BA	0.93	C4D—H4DA	0.93
C5B—C6B	1.409 (3)	C5D—C6D	1.406 (3)
C5B—H5BA	0.93	C5D—H5DA	0.93
C6B—C7B	1.444 (3)	C6D—C7D	1.450 (3)
C7B—H7BA	0.93	C7D—H7DA	0.93
C8B—C9B	1.392 (3)	C8D—C9D	1.387 (3)
C8B—C13B	1.402 (3)	C8D—C13D	1.399 (3)
C9B—C10B	1.379 (3)	C9D—C10D	1.371 (4)
C9B—H9BA	0.93	C9D—H9DA	0.93
C10B—C11B	1.394 (4)	C10D—C11D	1.383 (4)
C10B—C21F	1.53 (3)	C10D—C21H	1.52 (6)
C10B—H10B	0.93	C10D—H10D	0.93
C11B—C12B	1.395 (3)	C11D—C12D	1.395 (3)
C11B—C21B	1.504 (3)	C11D—C21D	1.507 (3)
C11B—H11B	0.93	C11D—H11D	0.93
C12B—C13B	1.392 (3)	C12D—C13D	1.390 (3)
C12B—H12B	0.93	C12D—H12D	0.93
C14B—C15B	1.409 (3)	C14D—C15D	1.397 (3)
C14B—H14B	0.93	C14D—H14D	0.93
C15B—C16B	1.423 (3)	C15D—C16D	1.423 (3)
C15B—C20B	1.430 (3)	C15D—C20D	1.429 (3)
C16B—C17B	1.359 (3)	C16D—C17D	1.361 (3)
C16B—H16B	0.93	C16D—H16D	0.93
C17B—C18B	1.408 (3)	C17D—C18D	1.406 (3)
C17B—H17B	0.93	C17D—H17D	0.93
C18B—C19B	1.363 (3)	C18D—C19D	1.359 (3)
C18B—H18B	0.93	C18D—H18D	0.93
C19B—C20B	1.426 (3)	C19D—C20D	1.431 (3)
C21B—H21D	0.96	C21D—H21J	0.96
C21B—H21E	0.96	C21D—H21K	0.96
C21B—H21F	0.96	C21D—H21L	0.96
C21F—H21S	0.96	C21H—H21V	0.96
C21F—H21T	0.96	C21H—H21W	0.96
C21F—H21U	0.96	C21H—H21X	0.96
			
C2A—O1A—H1OA	117 (2)	C2C—O1C—H1OC	114 (2)
C1A—O2A—H2OA	106 (2)	C1C—O2C—H2OC	104.0 (18)
C19A—O4A—H4OA	111 (3)	C19C—O4C—H4OC	107 (2)
C7A—N1A—C8A	118.8 (2)	C7C—N1C—C8C	119.7 (2)
C14A—N2A—C13A	126.7 (2)	C14C—N2C—C13C	126.9 (2)
C14A—N2A—H2NA	112.6 (18)	C14C—N2C—H2NC	113.5 (15)
C13A—N2A—H2NA	120.6 (18)	C13C—N2C—H2NC	119.6 (15)
O2A—C1A—C2A	116.8 (2)	O2C—C1C—C2C	117.1 (2)
O2A—C1A—C6A	123.5 (2)	O2C—C1C—C6C	122.6 (2)
C2A—C1A—C6A	119.7 (2)	C2C—C1C—C6C	120.2 (2)
O1A—C2A—C3A	119.5 (2)	O1C—C2C—C3C	119.5 (2)
O1A—C2A—C1A	120.5 (2)	O1C—C2C—C1C	120.9 (2)
C3A—C2A—C1A	119.9 (2)	C3C—C2C—C1C	119.5 (2)
C2A—C3A—C4A	120.6 (2)	C2C—C3C—C4C	120.7 (2)
C2A—C3A—H3AA	119.7	C2C—C3C—H3CA	119.7
C4A—C3A—H3AA	119.7	C4C—C3C—H3CA	119.7
C5A—C4A—C3A	120.0 (2)	C5C—C4C—C3C	120.3 (2)
C5A—C4A—H4AA	120.0	C5C—C4C—H4CA	119.8
C3A—C4A—H4AA	120.0	C3C—C4C—H4CA	119.8
C4A—C5A—C6A	120.4 (2)	C4C—C5C—C6C	120.1 (2)
C4A—C5A—H5AA	119.8	C4C—C5C—H5CA	119.9
C6A—C5A—H5AA	119.8	C6C—C5C—H5CA	119.9
C1A—C6A—C5A	119.4 (2)	C1C—C6C—C5C	119.1 (2)
C1A—C6A—C7A	121.5 (2)	C1C—C6C—C7C	121.2 (2)
C5A—C6A—C7A	119.1 (2)	C5C—C6C—C7C	119.7 (2)
N1A—C7A—C6A	123.8 (2)	N1C—C7C—C6C	122.6 (2)
N1A—C7A—H7AA	118.1	N1C—C7C—H7CA	118.7
C6A—C7A—H7AA	118.1	C6C—C7C—H7CA	118.7
C13A—C8A—C9A	117.8 (2)	C9C—C8C—C13C	118.3 (2)
C13A—C8A—N1A	117.8 (2)	C9C—C8C—N1C	123.6 (2)
C9A—C8A—N1A	124.3 (2)	C13C—C8C—N1C	118.0 (2)
C10A—C9A—C8A	121.3 (3)	C10C—C9C—C8C	121.3 (2)
C10A—C9A—H9AA	119.3	C10C—C9C—H9CA	119.4
C8A—C9A—H9AA	119.3	C8C—C9C—H9CA	119.4
C21E—C10A—C9A	132.1 (8)	C9C—C10C—C11C	120.5 (2)
C21E—C10A—C11A	106.8 (8)	C9C—C10C—C21G	128.2 (5)
C9A—C10A—C11A	120.9 (3)	C11C—C10C—C21G	110.5 (5)
C9A—C10A—H10A	119.6	C9C—C10C—H10C	119.7
C11A—C10A—H10A	119.6	C11C—C10C—H10C	119.7
C10A—C11A—C12A	118.9 (2)	C10C—C11C—C12C	119.0 (2)
C10A—C11A—C21A	119.4 (2)	C10C—C11C—C21C	118.8 (3)
C12A—C11A—C21A	121.7 (3)	C12C—C11C—C21C	122.0 (3)
C10A—C11A—H11A	120.5	C10C—C11C—H11C	120.5
C12A—C11A—H11A	120.5	C12C—C11C—H11C	120.5
C11A—C12A—C13A	120.8 (3)	C11C—C12C—C13C	120.6 (2)
C11A—C12A—H12A	119.6	C11C—C12C—H12C	119.7
C13A—C12A—H12A	119.6	C13C—C12C—H12C	119.7
C8A—C13A—C12A	120.2 (2)	C12C—C13C—C8C	120.2 (2)
C8A—C13A—N2A	118.7 (2)	C12C—C13C—N2C	122.4 (2)
C12A—C13A—N2A	121.0 (2)	C8C—C13C—N2C	117.4 (2)
N2A—C14A—C15A	124.0 (2)	N2C—C14C—C15C	123.5 (2)
N2A—C14A—H14A	118.0	N2C—C14C—H14C	118.3
C15A—C14A—H14A	118.0	C15C—C14C—H14C	118.3
C14A—C15A—C16A	119.7 (2)	C14C—C15C—C16C	119.0 (2)
C14A—C15A—C20A	120.3 (2)	C14C—C15C—C20C	120.5 (2)
C16A—C15A—C20A	120.0 (2)	C16C—C15C—C20C	120.5 (2)
C17A—C16A—C15A	120.9 (3)	C17C—C16C—C15C	120.2 (2)
C17A—C16A—H16A	119.6	C17C—C16C—H16C	119.9
C15A—C16A—H16A	119.6	C15C—C16C—H16C	119.9
C16A—C17A—C18A	120.5 (3)	C16C—C17C—C18C	120.3 (2)
C16A—C17A—H17A	119.8	C16C—C17C—H17C	119.9
C18A—C17A—H17A	119.8	C18C—C17C—H17C	119.9
C19A—C18A—C17A	120.1 (3)	C19C—C18C—C17C	121.1 (2)
C19A—C18A—H18A	120.0	C19C—C18C—H18C	119.4
C17A—C18A—H18A	120.0	C17C—C18C—H18C	119.4
C18A—C19A—O4A	120.7 (3)	C18C—C19C—O4C	120.7 (2)
C18A—C19A—C20A	121.7 (3)	C18C—C19C—C20C	120.9 (2)
O4A—C19A—C20A	117.6 (2)	O4C—C19C—C20C	118.4 (2)
O3A—C20A—C19A	120.1 (2)	O3C—C20C—C15C	123.0 (2)
O3A—C20A—C15A	123.1 (2)	O3C—C20C—C19C	120.0 (2)
C19A—C20A—C15A	116.8 (2)	C15C—C20C—C19C	117.0 (2)
C11A—C21A—H21A	109.5	C11C—C21C—H21G	109.5
C11A—C21A—H21B	109.5	C11C—C21C—H21H	109.5
C11A—C21A—H21C	109.5	C11C—C21C—H21I	109.5
C10A—C21E—H21M	109.5	C10C—C21G—H21P	109.5
C10A—C21E—H21N	109.5	C10C—C21G—H21Q	109.5
H21M—C21E—H21N	109.5	H21P—C21G—H21Q	109.5
C10A—C21E—H21O	109.5	C10C—C21G—H21R	109.5
H21M—C21E—H21O	109.5	H21P—C21G—H21R	109.5
H21N—C21E—H21O	109.5	H21Q—C21G—H21R	109.5
C2B—O1B—H1OB	112.4 (19)	C2D—O1D—H1OD	108 (2)
C1B—O2B—H2OB	107.6 (17)	C1D—O2D—H2OD	106.8 (18)
C19B—O4B—H4OB	109 (2)	C19D—O4D—H4OD	110 (2)
C7B—N1B—C8B	118.75 (19)	C7D—N1D—C8D	119.4 (2)
C14B—N2B—C13B	127.8 (2)	C14D—N2D—C13D	127.0 (2)
C14B—N2B—H2NB	109.9 (17)	C14D—N2D—H2ND	111.9 (16)
C13B—N2B—H2NB	122.0 (17)	C13D—N2D—H2ND	120.9 (16)
O2B—C1B—C2B	117.1 (2)	O2D—C1D—C2D	116.5 (2)
O2B—C1B—C6B	122.6 (2)	O2D—C1D—C6D	123.0 (2)
C2B—C1B—C6B	120.3 (2)	C2D—C1D—C6D	120.5 (2)
O1B—C2B—C3B	119.1 (2)	O1D—C2D—C3D	119.3 (2)
O1B—C2B—C1B	121.6 (2)	O1D—C2D—C1D	121.1 (2)
C3B—C2B—C1B	119.3 (2)	C3D—C2D—C1D	119.6 (2)
C2B—C3B—C4B	121.0 (2)	C2D—C3D—C4D	120.3 (2)
C2B—C3B—H3BA	119.5	C2D—C3D—H3DA	119.9
C4B—C3B—H3BA	119.5	C4D—C3D—H3DA	119.9
C5B—C4B—C3B	120.1 (2)	C5D—C4D—C3D	120.3 (3)
C5B—C4B—H4BA	120.0	C5D—C4D—H4DA	119.8
C3B—C4B—H4BA	120.0	C3D—C4D—H4DA	119.8
C4B—C5B—C6B	120.3 (2)	C4D—C5D—C6D	120.7 (2)
C4B—C5B—H5BA	119.8	C4D—C5D—H5DA	119.7
C6B—C5B—H5BA	119.8	C6D—C5D—H5DA	119.7
C1B—C6B—C5B	119.0 (2)	C1D—C6D—C5D	118.6 (2)
C1B—C6B—C7B	121.8 (2)	C1D—C6D—C7D	120.9 (2)
C5B—C6B—C7B	119.2 (2)	C5D—C6D—C7D	120.4 (2)
N1B—C7B—C6B	123.5 (2)	N1D—C7D—C6D	123.2 (2)
N1B—C7B—H7BA	118.2	N1D—C7D—H7DA	118.4
C6B—C7B—H7BA	118.2	C6D—C7D—H7DA	118.4
C9B—C8B—C13B	118.6 (2)	C9D—C8D—C13D	117.9 (2)
C9B—C8B—N1B	123.7 (2)	C9D—C8D—N1D	124.4 (2)
C13B—C8B—N1B	117.6 (2)	C13D—C8D—N1D	117.7 (2)
C10B—C9B—C8B	120.5 (2)	C10D—C9D—C8D	121.2 (3)
C10B—C9B—H9BA	119.7	C10D—C9D—H9DA	119.4
C8B—C9B—H9BA	119.7	C8D—C9D—H9DA	119.4
C9B—C10B—C11B	121.3 (2)	C9D—C10D—C11D	121.4 (3)
C9B—C10B—C21F	139.3 (11)	C9D—C10D—C21H	147 (3)
C11B—C10B—C21F	94.8 (11)	C11D—C10D—C21H	91 (3)
C9B—C10B—H10B	119.3	C9D—C10D—H10D	119.3
C11B—C10B—H10B	119.3	C11D—C10D—H10D	119.3
C10B—C11B—C12B	118.5 (2)	C10D—C11D—C12D	118.5 (2)
C10B—C11B—C21B	121.0 (2)	C10D—C11D—C21D	121.3 (2)
C12B—C11B—C21B	120.4 (2)	C12D—C11D—C21D	120.2 (3)
C10B—C11B—H11B	120.7	C10D—C11D—H11D	120.8
C12B—C11B—H11B	120.7	C12D—C11D—H11D	120.8
C13B—C12B—C11B	120.4 (2)	C13D—C12D—C11D	120.2 (2)
C13B—C12B—H12B	119.8	C13D—C12D—H12D	119.9
C11B—C12B—H12B	119.8	C11D—C12D—H12D	119.9
C12B—C13B—C8B	120.5 (2)	C12D—C13D—C8D	120.9 (2)
C12B—C13B—N2B	122.2 (2)	C12D—C13D—N2D	121.9 (2)
C8B—C13B—N2B	117.25 (19)	C8D—C13D—N2D	117.2 (2)
N2B—C14B—C15B	122.7 (2)	N2D—C14D—C15D	124.1 (2)
N2B—C14B—H14B	118.6	N2D—C14D—H14D	118.0
C15B—C14B—H14B	118.6	C15D—C14D—H14D	118.0
C14B—C15B—C16B	119.8 (2)	C14D—C15D—C16D	119.6 (2)
C14B—C15B—C20B	119.8 (2)	C14D—C15D—C20D	120.4 (2)
C16B—C15B—C20B	120.4 (2)	C16D—C15D—C20D	120.1 (2)
C17B—C16B—C15B	120.5 (2)	C17D—C16D—C15D	120.4 (2)
C17B—C16B—H16B	119.8	C17D—C16D—H16D	119.8
C15B—C16B—H16B	119.8	C15D—C16D—H16D	119.8
C16B—C17B—C18B	119.9 (2)	C16D—C17D—C18D	120.1 (2)
C16B—C17B—H17B	120.0	C16D—C17D—H17D	120.0
C18B—C17B—H17B	120.0	C18D—C17D—H17D	120.0
C19B—C18B—C17B	121.1 (2)	C19D—C18D—C17D	121.3 (2)
C19B—C18B—H18B	119.5	C19D—C18D—H18D	119.4
C17B—C18B—H18B	119.5	C17D—C18D—H18D	119.4
C18B—C19B—O4B	120.6 (2)	C18D—C19D—O4D	120.6 (2)
C18B—C19B—C20B	121.5 (2)	C18D—C19D—C20D	121.1 (2)
O4B—C19B—C20B	117.9 (2)	O4D—C19D—C20D	118.3 (2)
O3B—C20B—C19B	119.8 (2)	O3D—C20D—C15D	123.1 (2)
O3B—C20B—C15B	123.6 (2)	O3D—C20D—C19D	119.9 (2)
C19B—C20B—C15B	116.7 (2)	C15D—C20D—C19D	117.0 (2)
C11B—C21B—H21D	109.5	C11D—C21D—H21J	109.5
C11B—C21B—H21E	109.5	C11D—C21D—H21K	109.5
C11B—C21B—H21F	109.5	C11D—C21D—H21L	109.5
C10B—C21F—H21S	109.5	C10D—C21H—H21V	109.5
C10B—C21F—H21T	109.5	C10D—C21H—H21W	109.5
H21S—C21F—H21T	109.5	H21V—C21H—H21W	109.5
C10B—C21F—H21U	109.5	C10D—C21H—H21X	109.5
H21S—C21F—H21U	109.5	H21V—C21H—H21X	109.5
H21T—C21F—H21U	109.5	H21W—C21H—H21X	109.5
			
O2A—C1A—C2A—O1A	0.2 (3)	O2C—C1C—C2C—O1C	1.9 (3)
C6A—C1A—C2A—O1A	−179.2 (2)	C6C—C1C—C2C—O1C	−176.6 (2)
O2A—C1A—C2A—C3A	178.5 (2)	O2C—C1C—C2C—C3C	−177.6 (2)
C6A—C1A—C2A—C3A	−0.8 (4)	C6C—C1C—C2C—C3C	3.8 (4)
O1A—C2A—C3A—C4A	178.4 (2)	O1C—C2C—C3C—C4C	178.3 (2)
C1A—C2A—C3A—C4A	0.1 (4)	C1C—C2C—C3C—C4C	−2.1 (4)
C2A—C3A—C4A—C5A	0.1 (4)	C2C—C3C—C4C—C5C	−0.7 (4)
C3A—C4A—C5A—C6A	0.5 (4)	C3C—C4C—C5C—C6C	1.8 (4)
O2A—C1A—C6A—C5A	−177.9 (2)	O2C—C1C—C6C—C5C	178.8 (2)
C2A—C1A—C6A—C5A	1.4 (4)	C2C—C1C—C6C—C5C	−2.7 (3)
O2A—C1A—C6A—C7A	2.9 (4)	O2C—C1C—C6C—C7C	−2.2 (4)
C2A—C1A—C6A—C7A	−177.8 (2)	C2C—C1C—C6C—C7C	176.3 (2)
C4A—C5A—C6A—C1A	−1.2 (4)	C4C—C5C—C6C—C1C	−0.1 (4)
C4A—C5A—C6A—C7A	178.0 (3)	C4C—C5C—C6C—C7C	−179.1 (2)
C8A—N1A—C7A—C6A	179.5 (2)	C8C—N1C—C7C—C6C	177.5 (2)
C1A—C6A—C7A—N1A	−1.1 (4)	C1C—C6C—C7C—N1C	−1.2 (4)
C5A—C6A—C7A—N1A	179.7 (2)	C5C—C6C—C7C—N1C	177.8 (2)
C7A—N1A—C8A—C13A	−161.8 (2)	C7C—N1C—C8C—C9C	−35.0 (3)
C7A—N1A—C8A—C9A	17.8 (4)	C7C—N1C—C8C—C13C	148.5 (2)
C13A—C8A—C9A—C10A	1.6 (4)	C13C—C8C—C9C—C10C	−0.2 (4)
N1A—C8A—C9A—C10A	−177.9 (2)	N1C—C8C—C9C—C10C	−176.6 (2)
C8A—C9A—C10A—C21E	−174.3 (10)	C8C—C9C—C10C—C11C	1.4 (4)
C8A—C9A—C10A—C11A	−1.1 (4)	C8C—C9C—C10C—C21G	−167.9 (6)
C21E—C10A—C11A—C12A	174.3 (8)	C9C—C10C—C11C—C12C	−1.3 (4)
C9A—C10A—C11A—C12A	−0.4 (4)	C21G—C10C—C11C—C12C	169.7 (6)
C21E—C10A—C11A—C21A	−6.2 (8)	C9C—C10C—C11C—C21C	174.2 (3)
C9A—C10A—C11A—C21A	179.1 (2)	C21G—C10C—C11C—C21C	−14.7 (6)
C10A—C11A—C12A—C13A	1.4 (4)	C10C—C11C—C12C—C13C	0.0 (4)
C21A—C11A—C12A—C13A	−178.1 (2)	C21C—C11C—C12C—C13C	−175.4 (3)
C9A—C8A—C13A—C12A	−0.6 (4)	C11C—C12C—C13C—C8C	1.2 (4)
N1A—C8A—C13A—C12A	178.9 (2)	C11C—C12C—C13C—N2C	−176.8 (2)
C9A—C8A—C13A—N2A	179.1 (2)	C9C—C8C—C13C—C12C	−1.1 (3)
N1A—C8A—C13A—N2A	−1.3 (3)	N1C—C8C—C13C—C12C	175.5 (2)
C11A—C12A—C13A—C8A	−0.9 (4)	C9C—C8C—C13C—N2C	177.0 (2)
C11A—C12A—C13A—N2A	179.4 (2)	N1C—C8C—C13C—N2C	−6.4 (3)
C14A—N2A—C13A—C8A	164.4 (2)	C14C—N2C—C13C—C12C	10.5 (4)
C14A—N2A—C13A—C12A	−15.9 (4)	C14C—N2C—C13C—C8C	−167.6 (2)
C13A—N2A—C14A—C15A	179.0 (2)	C13C—N2C—C14C—C15C	179.7 (2)
N2A—C14A—C15A—C16A	−177.8 (2)	N2C—C14C—C15C—C16C	178.5 (2)
N2A—C14A—C15A—C20A	2.2 (4)	N2C—C14C—C15C—C20C	0.4 (4)
C14A—C15A—C16A—C17A	−179.7 (2)	C14C—C15C—C16C—C17C	−177.3 (2)
C20A—C15A—C16A—C17A	0.2 (4)	C20C—C15C—C16C—C17C	0.8 (4)
C15A—C16A—C17A—C18A	1.2 (4)	C15C—C16C—C17C—C18C	0.4 (4)
C16A—C17A—C18A—C19A	−0.7 (4)	C16C—C17C—C18C—C19C	−1.2 (4)
C17A—C18A—C19A—O4A	179.1 (3)	C17C—C18C—C19C—O4C	179.5 (2)
C17A—C18A—C19A—C20A	−1.3 (4)	C17C—C18C—C19C—C20C	0.8 (4)
C18A—C19A—C20A—O3A	−177.4 (3)	C14C—C15C—C20C—O3C	−2.2 (4)
O4A—C19A—C20A—O3A	2.2 (4)	C16C—C15C—C20C—O3C	179.6 (2)
C18A—C19A—C20A—C15A	2.7 (4)	C14C—C15C—C20C—C19C	177.0 (2)
O4A—C19A—C20A—C15A	−177.8 (2)	C16C—C15C—C20C—C19C	−1.1 (3)
C14A—C15A—C20A—O3A	−2.1 (4)	C18C—C19C—C20C—O3C	179.6 (2)
C16A—C15A—C20A—O3A	178.0 (2)	O4C—C19C—C20C—O3C	0.8 (3)
C14A—C15A—C20A—C19A	177.9 (2)	C18C—C19C—C20C—C15C	0.3 (3)
C16A—C15A—C20A—C19A	−2.1 (4)	O4C—C19C—C20C—C15C	−178.4 (2)
O2B—C1B—C2B—O1B	0.9 (3)	O2D—C1D—C2D—O1D	0.0 (3)
C6B—C1B—C2B—O1B	−179.0 (2)	C6D—C1D—C2D—O1D	−178.9 (2)
O2B—C1B—C2B—C3B	−177.3 (2)	O2D—C1D—C2D—C3D	−179.1 (2)
C6B—C1B—C2B—C3B	2.8 (3)	C6D—C1D—C2D—C3D	2.0 (3)
O1B—C2B—C3B—C4B	179.6 (2)	O1D—C2D—C3D—C4D	−178.4 (2)
C1B—C2B—C3B—C4B	−2.2 (4)	C1D—C2D—C3D—C4D	0.7 (4)
C2B—C3B—C4B—C5B	0.2 (4)	C2D—C3D—C4D—C5D	−2.0 (4)
C3B—C4B—C5B—C6B	1.2 (4)	C3D—C4D—C5D—C6D	0.6 (4)
O2B—C1B—C6B—C5B	178.6 (2)	O2D—C1D—C6D—C5D	177.8 (2)
C2B—C1B—C6B—C5B	−1.5 (3)	C2D—C1D—C6D—C5D	−3.4 (3)
O2B—C1B—C6B—C7B	0.3 (4)	O2D—C1D—C6D—C7D	−6.5 (4)
C2B—C1B—C6B—C7B	−179.8 (2)	C2D—C1D—C6D—C7D	172.2 (2)
C4B—C5B—C6B—C1B	−0.5 (3)	C4D—C5D—C6D—C1D	2.1 (4)
C4B—C5B—C6B—C7B	177.8 (2)	C4D—C5D—C6D—C7D	−173.6 (2)
C8B—N1B—C7B—C6B	175.9 (2)	C8D—N1D—C7D—C6D	−176.6 (2)
C1B—C6B—C7B—N1B	−0.4 (4)	C1D—C6D—C7D—N1D	−2.2 (4)
C5B—C6B—C7B—N1B	−178.7 (2)	C5D—C6D—C7D—N1D	173.4 (2)
C7B—N1B—C8B—C9B	−29.9 (3)	C7D—N1D—C8D—C9D	−37.0 (4)
C7B—N1B—C8B—C13B	151.4 (2)	C7D—N1D—C8D—C13D	144.6 (2)
C13B—C8B—C9B—C10B	−1.4 (3)	C13D—C8D—C9D—C10D	−1.7 (4)
N1B—C8B—C9B—C10B	179.9 (2)	N1D—C8D—C9D—C10D	180.0 (2)
C8B—C9B—C10B—C11B	1.8 (4)	C8D—C9D—C10D—C11D	0.4 (4)
C8B—C9B—C10B—C21F	−147.3 (17)	C8D—C9D—C10D—C21H	−169 (5)
C9B—C10B—C11B—C12B	−0.7 (4)	C9D—C10D—C11D—C12D	0.5 (4)
C21F—C10B—C11B—C12B	159.6 (11)	C21H—C10D—C11D—C12D	175 (2)
C9B—C10B—C11B—C21B	178.9 (2)	C9D—C10D—C11D—C21D	−178.8 (3)
C21F—C10B—C11B—C21B	−20.8 (11)	C21H—C10D—C11D—C21D	−4(2)
C10B—C11B—C12B—C13B	−0.6 (3)	C10D—C11D—C12D—C13D	0.0 (4)
C21B—C11B—C12B—C13B	179.8 (2)	C21D—C11D—C12D—C13D	179.3 (2)
C11B—C12B—C13B—C8B	0.9 (3)	C11D—C12D—C13D—C8D	−1.3 (4)
C11B—C12B—C13B—N2B	−177.2 (2)	C11D—C12D—C13D—N2D	178.2 (2)
C9B—C8B—C13B—C12B	0.1 (3)	C9D—C8D—C13D—C12D	2.2 (4)
N1B—C8B—C13B—C12B	178.9 (2)	N1D—C8D—C13D—C12D	−179.4 (2)
C9B—C8B—C13B—N2B	178.3 (2)	C9D—C8D—C13D—N2D	−177.4 (2)
N1B—C8B—C13B—N2B	−2.9 (3)	N1D—C8D—C13D—N2D	1.1 (3)
C14B—N2B—C13B—C12B	4.6 (4)	C14D—N2D—C13D—C12D	−3.8 (4)
C14B—N2B—C13B—C8B	−173.6 (2)	C14D—N2D—C13D—C8D	175.8 (2)
C13B—N2B—C14B—C15B	176.3 (2)	C13D—N2D—C14D—C15D	−177.5 (2)
N2B—C14B—C15B—C16B	−179.0 (2)	N2D—C14D—C15D—C16D	−179.9 (2)
N2B—C14B—C15B—C20B	−1.4 (3)	N2D—C14D—C15D—C20D	−0.5 (4)
C14B—C15B—C16B—C17B	177.6 (2)	C14D—C15D—C16D—C17D	179.2 (2)
C20B—C15B—C16B—C17B	0.0 (4)	C20D—C15D—C16D—C17D	−0.2 (4)
C15B—C16B—C17B—C18B	0.4 (4)	C15D—C16D—C17D—C18D	0.5 (4)
C16B—C17B—C18B—C19B	−0.5 (4)	C16D—C17D—C18D—C19D	0.2 (4)
C17B—C18B—C19B—O4B	−179.6 (2)	C17D—C18D—C19D—O4D	179.2 (2)
C17B—C18B—C19B—C20B	0.2 (4)	C17D—C18D—C19D—C20D	−1.2 (4)
C18B—C19B—C20B—O3B	−178.7 (2)	C14D—C15D—C20D—O3D	−0.2 (4)
O4B—C19B—C20B—O3B	1.1 (3)	C16D—C15D—C20D—O3D	179.2 (2)
C18B—C19B—C20B—C15B	0.2 (3)	C14D—C15D—C20D—C19D	179.8 (2)
O4B—C19B—C20B—C15B	180.0 (2)	C16D—C15D—C20D—C19D	−0.8 (3)
C14B—C15B—C20B—O3B	0.9 (3)	C18D—C19D—C20D—O3D	−178.5 (2)
C16B—C15B—C20B—O3B	178.5 (2)	O4D—C19D—C20D—O3D	1.1 (3)
C14B—C15B—C20B—C19B	−177.9 (2)	C18D—C19D—C20D—C15D	1.5 (3)
C16B—C15B—C20B—C19B	−0.3 (3)	O4D—C19D—C20D—C15D	−178.9 (2)

### Hydrogen-bond geometry (Å, °)

**Table:**

*D*—H···*A*	*D*—H	H···*A*	*D*···*A*	*D*—H···*A*
O2A—H2OA···N1A	0.92 (3)	1.86 (3)	2.686 (3)	149 (3)
O2B—H2OB···N1B	0.96 (3)	1.82 (3)	2.671 (2)	146 (2)
O2C—H2OC···N1C	0.99 (3)	1.72 (3)	2.634 (3)	151 (3)
O2D—H2OD···N1D	0.95 (3)	1.81 (3)	2.652 (3)	147 (3)
N2A—H2NA···O3A	0.92 (3)	1.86 (3)	2.631 (3)	140 (2)
N2B—H2NB···O3B	1.03 (3)	1.70 (3)	2.596 (2)	144 (3)
N2C—H2NC···O3C	0.95 (3)	1.83 (2)	2.620 (3)	139 (2)
N2D—H2ND···O3D	0.97 (3)	1.80 (3)	2.624 (2)	140 (2)
O1C—H1OC···O3D	0.88 (3)	1.91 (4)	2.734 (3)	155 (3)
O1D—H1OD···O3C	0.91 (3)	1.93 (3)	2.724 (2)	146 (3)
O4C—H4OC···O2D	0.88 (4)	2.48 (4)	3.079 (3)	126 (3)
O4C—H4OC···O3D	0.88 (4)	2.31 (4)	3.026 (2)	138 (3)
O4D—H4OD···O2C	0.85 (3)	2.34 (3)	2.942 (2)	129 (3)
O4D—H4OD···O3C	0.85 (3)	2.29 (3)	2.943 (3)	134 (3)
O1A—H1OA···O3B^i^	0.89 (3)	1.88 (3)	2.732 (2)	160 (3)
O1B—H1OB···O3A^ii^	0.85 (3)	2.01 (3)	2.809 (3)	157 (3)
O4A—H4OA···O2B^i^	0.81 (4)	2.25 (4)	2.929 (3)	142 (4)
O4B—H4OB···O2A^ii^	0.91 (4)	2.33 (3)	3.021 (3)	133 (3)
O4B—H4OB···O3A^ii^	0.91 (4)	2.53 (4)	3.231 (3)	134 (3)
C5B—H5BA···O1D^iii^	0.93	2.52	3.372 (3)	152
C7B—H7BA···O1D^iii^	0.93	2.57	3.416 (3)	151
C14A—H14A···O4D^iv^	0.93	2.36	3.281 (3)	170
C14B—H14B···O1C^iv^	0.93	2.40	3.260 (3)	154
C14C—H14C···O4B^v^	0.93	2.46	3.323 (3)	154
C10D—H10D···Cg1^vi^	0.93	2.97	3.623 (3)	128
C9A—H9AA···Cg2^vii^	0.93	2.97	3.716 (3)	138
C9C—H9CA···Cg3	0.93	2.65	3.480 (3)	148
C21C—H21I···Cg4^v^	0.96	2.95	3.696 (4)	136
C4B—H4BA···Cg5^viii^	0.93	2.83	3.573 (3)	138
C17D—H17D···Cg6^ix^	0.93	2.69	3.450 (3)	139

Symmetry codes: (i) *x*+1, *y*, *z*; (ii) *x*−1, *y*, *z*; (iii) *x*, −*y*+3/2, *z*+1/2; (iv) −*x*+1, −*y*+1, −*z*+1; (v) −*x*, −*y*+1, −*z*+1; (vi) *x*, −*y*+1/2, *z*−3/2; (vii) −*x*+1, −*y*+1, −*z*+2;  (viii) *x*, −*y*+1/2, *z*−1/2; (ix) −*x*+1, *y*−1/2, −*z*+1/2.
